# Supplementary figures and images for: Increased prostaglandin-D2 in male STAT3-deficient hearts shifts cardiac progenitor cells from endothelial to white adipocyte differentiation
Source: PLoS Biol. 2020 Dec 28;18(12):e3000739. doi: 10.1371/journal.pbio.3000739 (PMC7793290; doi:10.1371/journal.pbio.3000739)

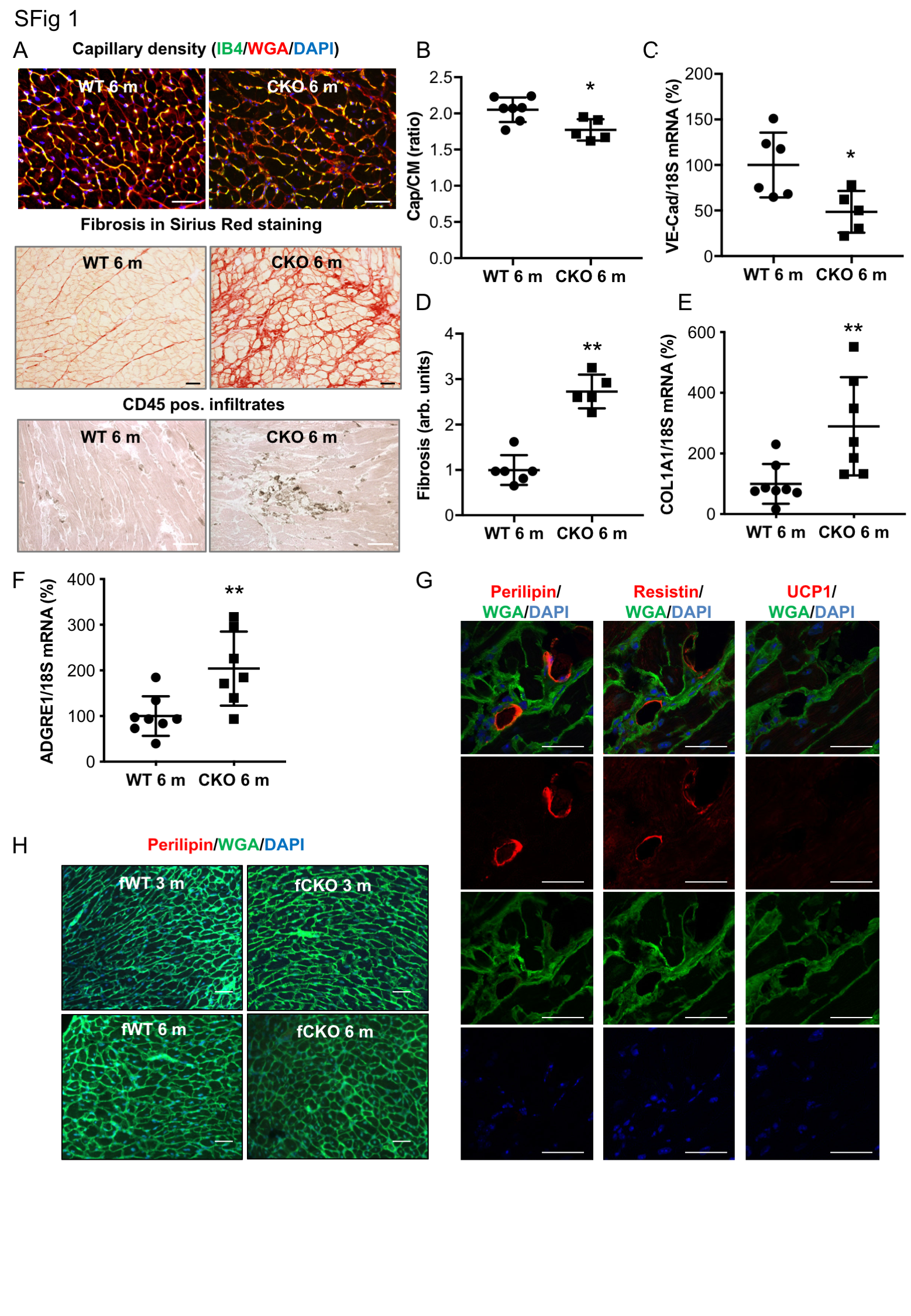

Supplement: S1 Fig — (A) Capillary density (upper panel: IB4 (green)/WGA (red) and nuclear DAPI staining (blue), Sirius Red staining (middle panel) and CD45 positive infiltrates (lower panel: brown, co-stained with eosin) of LV cryosections of 6 m male WT or CKO mice, scale bars: 50 μm. (B) Capillary density determined as the ratio of capillaries to CMs in transversely sectioned male WT (n = 7) and CKO (n = 5) LVs. (C) Dot plot summarizes VE-cadherin mRNA levels, of male WT (n = 6) and CKO LVs (n = 5), mean of WT was set at 100%. (D) Quantification of fibrosis from WT (n = 6) and CKO (n = 5) LVs in arbitrary units (arb. units). (E) Dot plot summarizes COL1A1 mRNA levels, of male WT (n = 8) and CKO LVs (n = 7), mean of WT was set at 100%. (F) Dot plot summarizes ADGRE1 mRNA levels of male WT (n = 8) and CKO LVs (n = 7), mean of WT was set at 100%. (G) Immunofluorescence staining of perilipin (red), resistin (red), or UCP-1 (red) counterstained with WGA-FITC (green) and DAPI (blue) in cryosections of heart tissue (male 6 m CKO mice), scale: 25 μM. (H) Perilipin staining in LV cryosections of 3- and 6-month-old (m) WT or CKO female (f) mice, perilipin (red), WGA (green), and DAPI (blue): scale bars: 50 μm. (B–F) All data are mean ± SD, * p <0.05, ** p <0.01 vs. WT, 2-tailed unpaired t test. Underlying data can be found in S1 Data. CKO, conditional knockout; CM, cardiomyocyte; FITC, fluorescein isothiocyanate; IB4, isolectin B4; LV, left ventricular; UCP-1, uncoupling protein 1; VE, vascular endothelial; WGA, wheat germ agglutinin; WT, wild-type. (TIF) [file pbio.3000739.s001.tif]

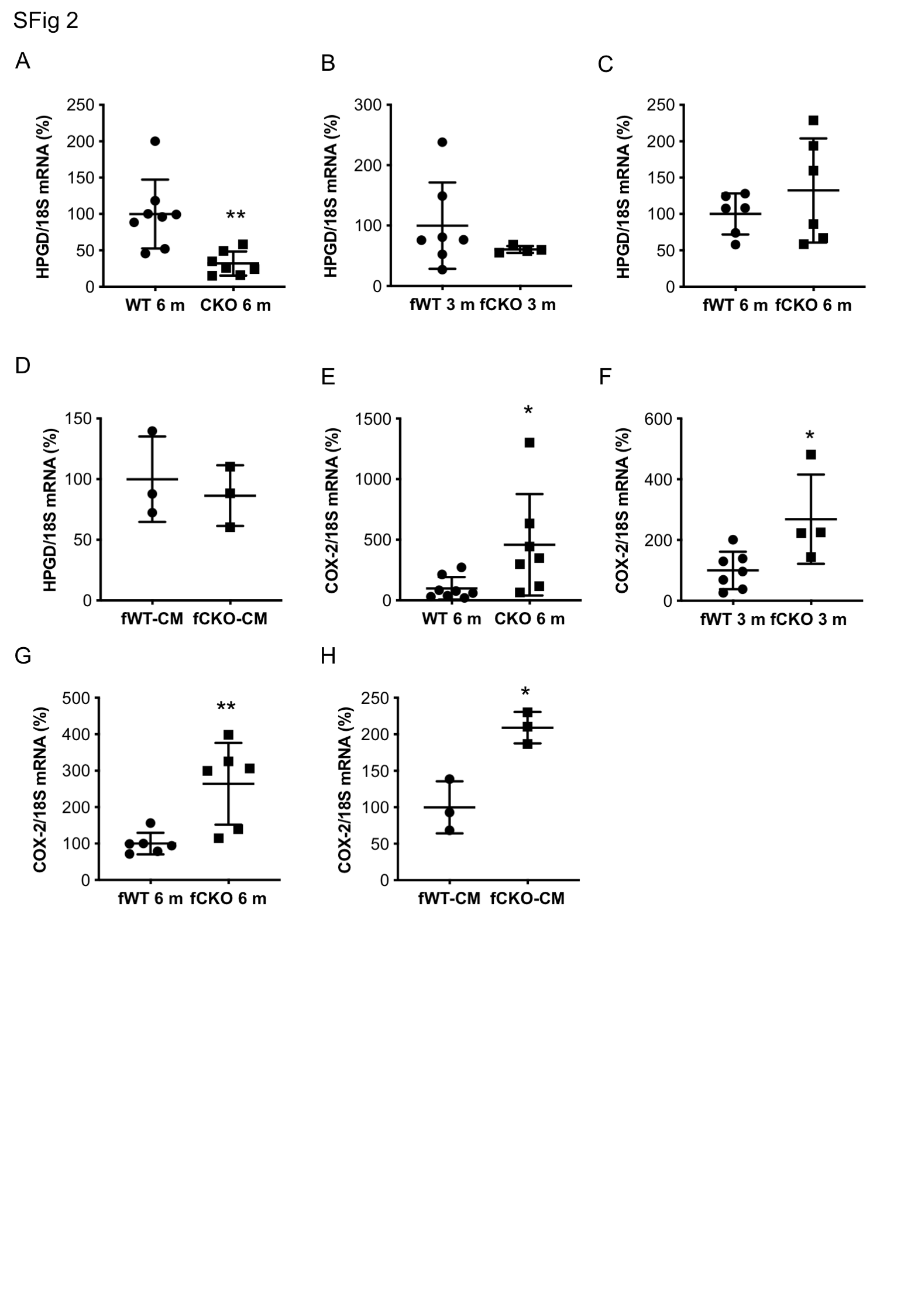

Supplement: S2 Fig — (A, E) Dot plots summarize mRNA levels of (A) HPGD and (E) COX-2 in LVs of 6-month-old male WT (n = 8) and CKO mice (n = 7). (B and F) Dot plots summarize mRNA levels of (B) HPGD and (F) COX-2 in LVs of 3-month-old female WT (n = 7) and CKO mice (n = 4). (C and G) Dot plots summarize mRNA levels of (C) HPGD and (G) COX-2 in LVs of 6-month-old female WT (n = 6) and CKO mice (n = 6). (D and H) Dot plots summarize (D) HPGD and (H) COX-2 mRNA levels of isolated adult female WT-CM and CKO-CM (CM isolated and pooled from 3 WT and 2 CKO mice). (A–H) All data are mean ± SD, and WT mean was set at 100%, * p < 0.05, ** p < 0.01 vs. WT, 2-tailed unpaired t tests. Underlying data can be found in S1 Data. CKO, conditional knockout; CM, cardiomyocyte; COX, cyclooxygenase; HPGD, hydroxyprostaglandin-dehydrogenase; LV, left ventricular; STAT3, signal transducer and activator of transcription factor-3; WT, wild-type. (TIF) [file pbio.3000739.s002.tif]

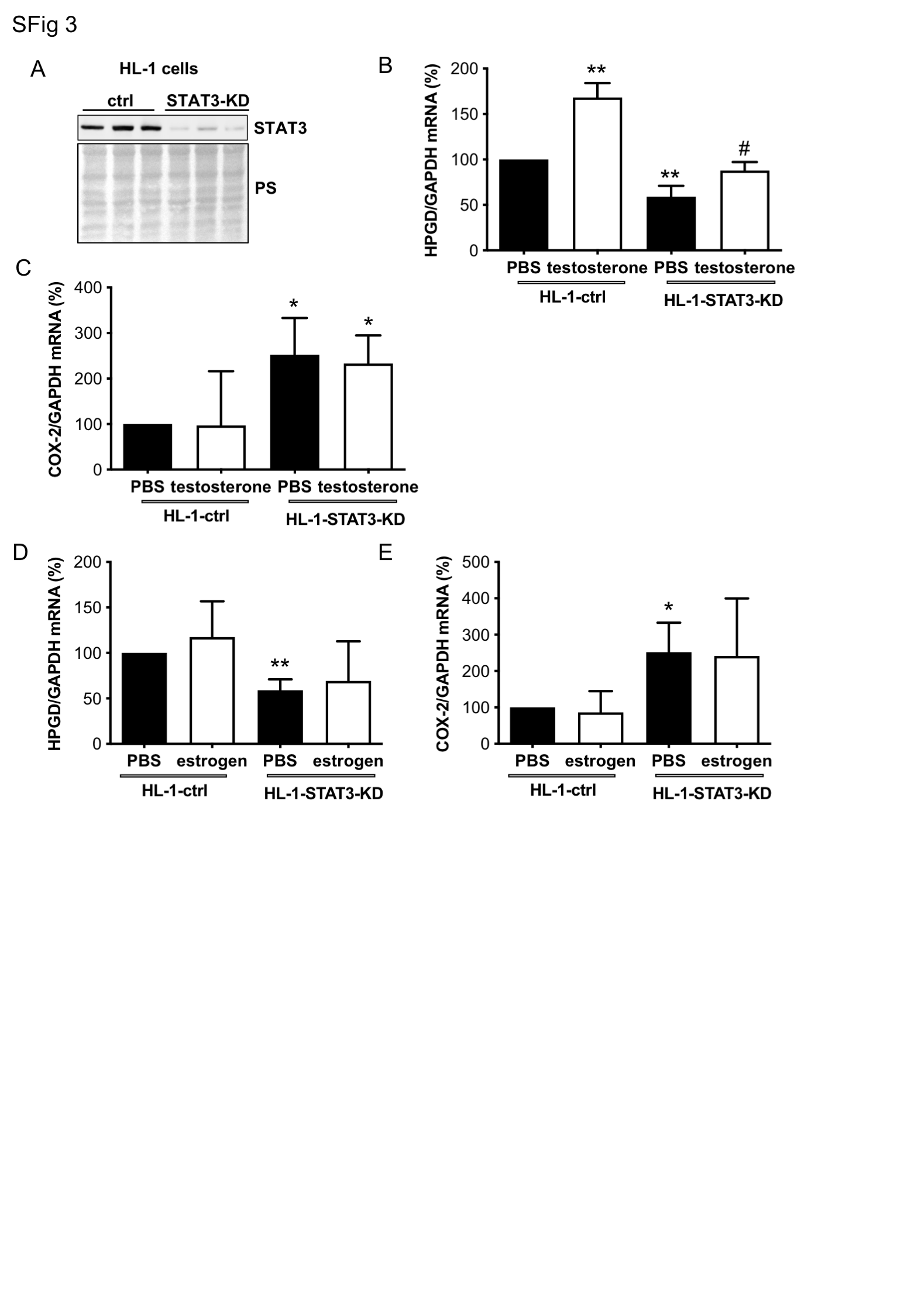

Supplement: S3 Fig — (A) Representative western blot showing protein expression of STAT3 in HL-1 control (ctrl) and STAT3-KD cells. PS served as a loading control. (B, C) Bar graphs summarize mRNA levels assessed by qRT-PCR of (B) HPGD and (C) COX-2 in HL-1 cells treated with testosterone (10 nM) for 24 h. (D, E) Bar graphs summarize mRNA levels assessed by qRT-PCR of (D) HPGD and (E) COX-2 in HL-1 cells treated with estrogen (10 nM) for 24 h. (B–E) Data are presented as mean ± SD, n = 4, and the mean of HL-1-ctrl PBS was set at 100%, * p <0.05, ** p <0.01 vs. HL-1-ctrl PBS, # p <0.05, ## p <0.01 vs. HL-1-STAT3-KD PBS, 2-way ANOVA with Bonferroni's multiple comparison test. Underlying data can be found in S1 Data and S11 Fig. COX, cyclooxygenase; HPGD, hydroxyprostaglandin-dehydrogenase; KD, knockdown; PS, Ponceau S; qRT-PCR, quantitative real-time PCR; STAT3, signal transducer and activator of transcription factor-3. (TIF) [file pbio.3000739.s003.tif]

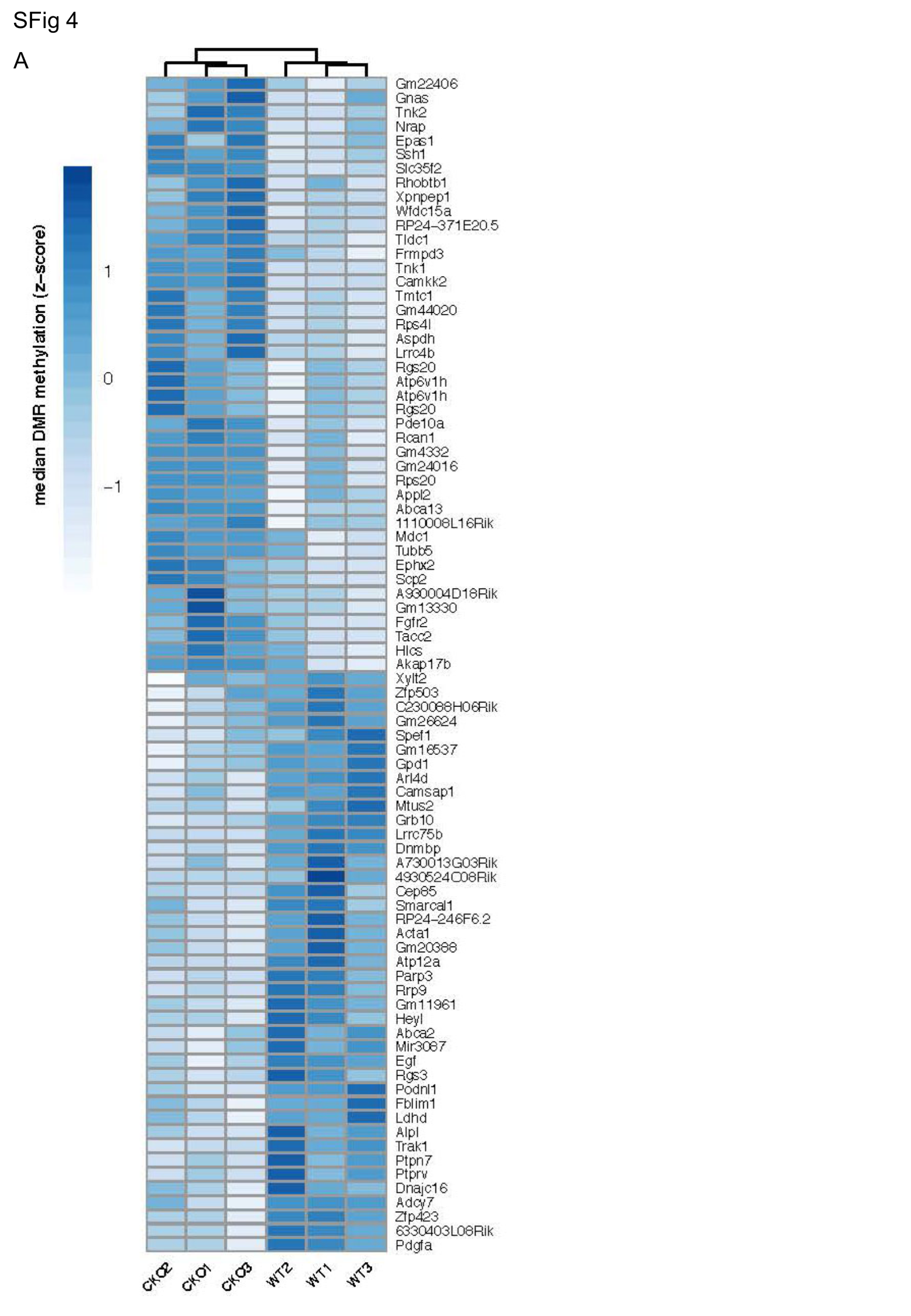

Supplement: S4 Fig — (A) Heatmap of median methylation values in predicted DMRs (n = 83) overlapping with 81 unique genes. Underlying data can be found in S1 Data. Sequencing data of the epigenetic analyses are available under accession number PRJNA602737 in the Sequence Read Archive. CKO, conditional knockout; CPC, cardiac progenitor cell; DMR, differentially methylated region; WT, wild-type. (TIF) [file pbio.3000739.s004.tif]

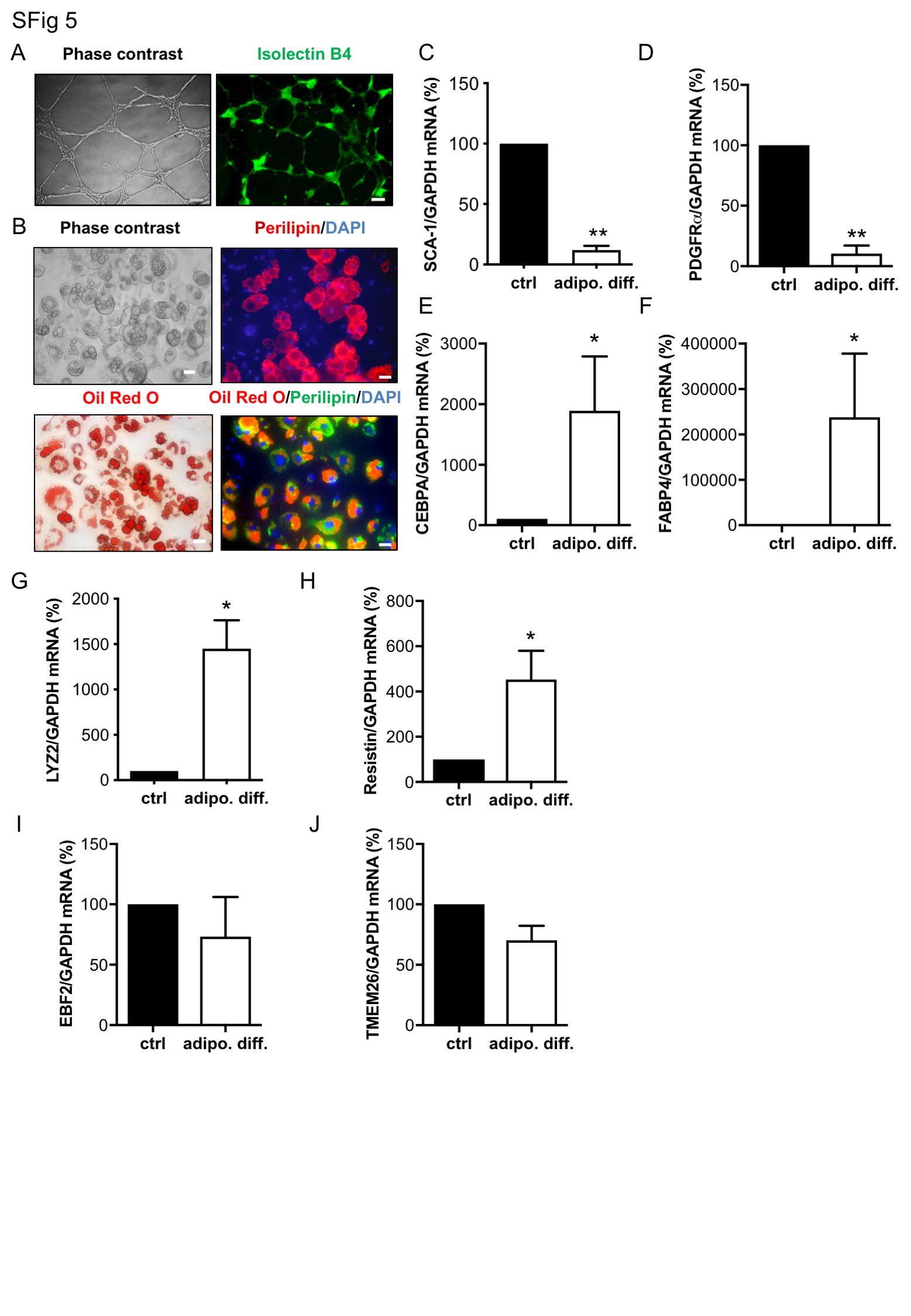

Supplement: S5 Fig — (A, B) Differentiation of cCPC expanded from single cells (A) on Matrigel (left panel: phase contrast, right panel: Isolectin B4 staining (green); scale bars indicate 100 μm) and (B) after adipogenic induction (upper left panel: phase contrast; upper right panel: perilipin staining [red], nuclear staining, DAPI [blue]; lower left panel: Oil Red O staining [red]; and lower right panel: perilipin staining [green], nuclear staining, DAPI [blue], Oil Red O staining [red]; scale bars indicate 50 μm). (C–J) mRNA levels of progenitor cell markers ((C) SCA1 and (D) PDGFRα), general adipocyte markers ((E) CEBPA, (F) FABP4), WAT markers ((G) LYZ2, (H) Resistin), and BAT/BET markers ((I) EBF2, (J) TMEM26) in undifferentiated and differentiated cCPC (n = 5 (C–F) and n = 3 (G–J) independent cell culture experiments. Statistically significant differences between the groups are represented as mean ± SD, and the mean of mRNA expression levels of undifferentiated cCPC were set at 100%, ** p <0.01 vs. control, * p < 0.05 vs. control, 1 sample t test). Underlying data can be found in S1 Data. cCPC, clonally expanded CPC; CEBPA, CCAAT/enhancer-binding protein alpha; CPC, cardiac progenitor cell; EBF2, early B cell factor 2; FABP4, fatty acid binding protein 4; PDGFRα, platelet-derived growth factor receptor alpha; TMEM26, transmembrane protein 26; WAT, white adipose tissue. (TIF) [file pbio.3000739.s005.tif]

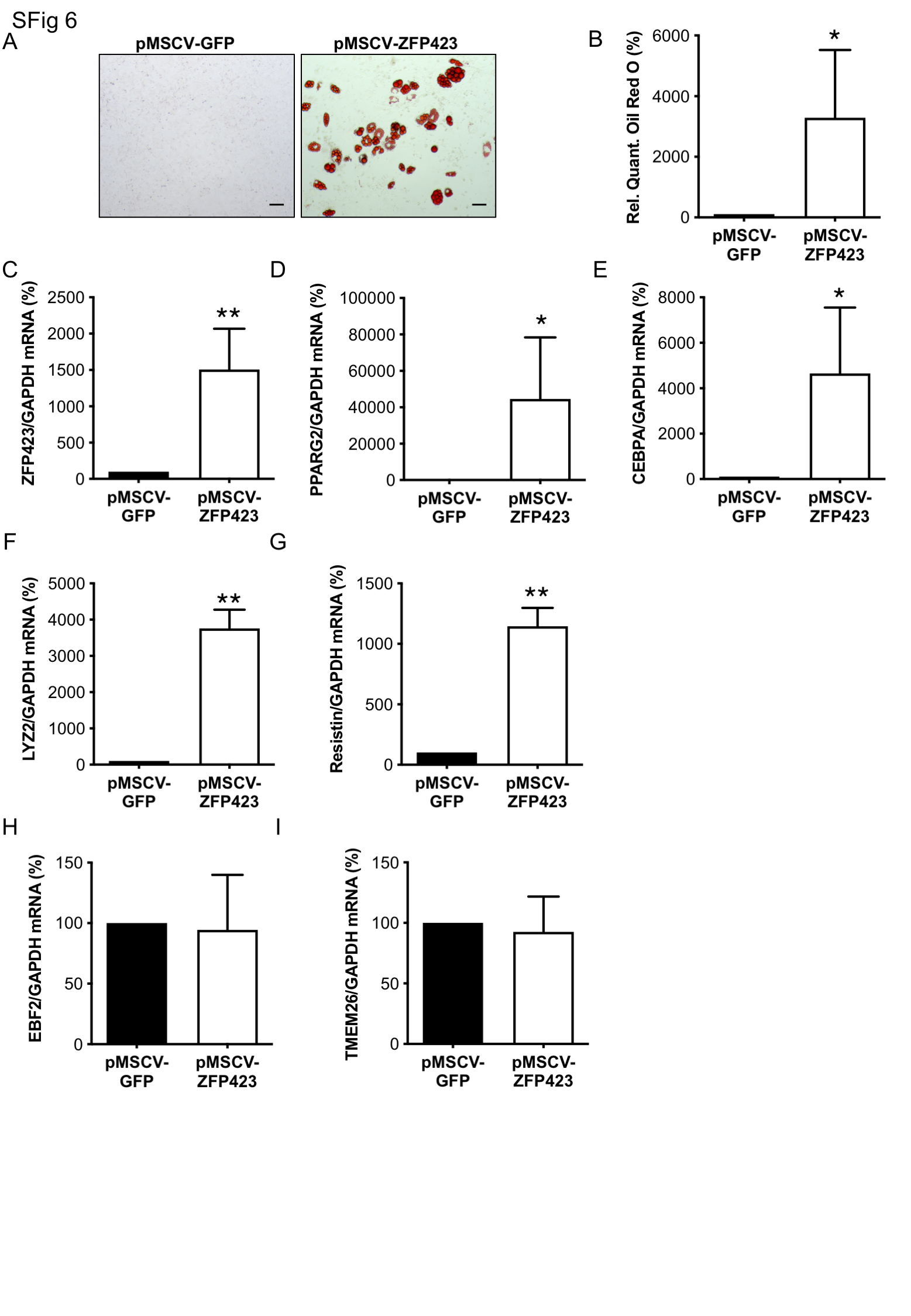

Supplement: S6 Fig — (A) Oil Red O staining of cCPC with retrovirally mediated overexpression of ZFP423. Control cells were transduced with the pMSCV-GFP control virusplasmid, scale bars: 50 μm. (B) Relative quantification of Oil Red O measured by absorbance at 492 nm. (C–E) qRT-PCR detects mRNA levels of (C) ZFP423 and of the adipocyte markers (D) CEBPA and (E) PPARγ. (F and G) qRT-PCR visualizes mRNA levels of the white adipocyte markers (F) LYZ2 and (G) resistin. (H and I) qRT-PCR visualizes mRNA levels of the brown/beige adipocyte markers (H) EBF2 and (I) TMEM26. (B–I) Data are presented as mean ± SD, and the mean of pMSCV-GFP transduced control cells was set to 100% (n = 6 (B–E) and n = 3 (F–I) independent experiments), * p < 0.05, ** p < 0.01 vs. pMSCV-GFP transduced control cells and 1 sample ttest. Underlying data can be found in S1 Data. cCPC, clonally expanded CPC; CEBPA, CCAAT/enhancer-binding protein alpha; CPC, cardiac progenitor cell; EBF2, early B cell factor 2; LYZ2, lysozyme 2; PPARγ, peroxisome proliferator-activated receptor gamma isoform; qRT-PCR, quantitative real-time PCR; TMEM26, transmembrane protein 26; (TIF) [file pbio.3000739.s006.tif]

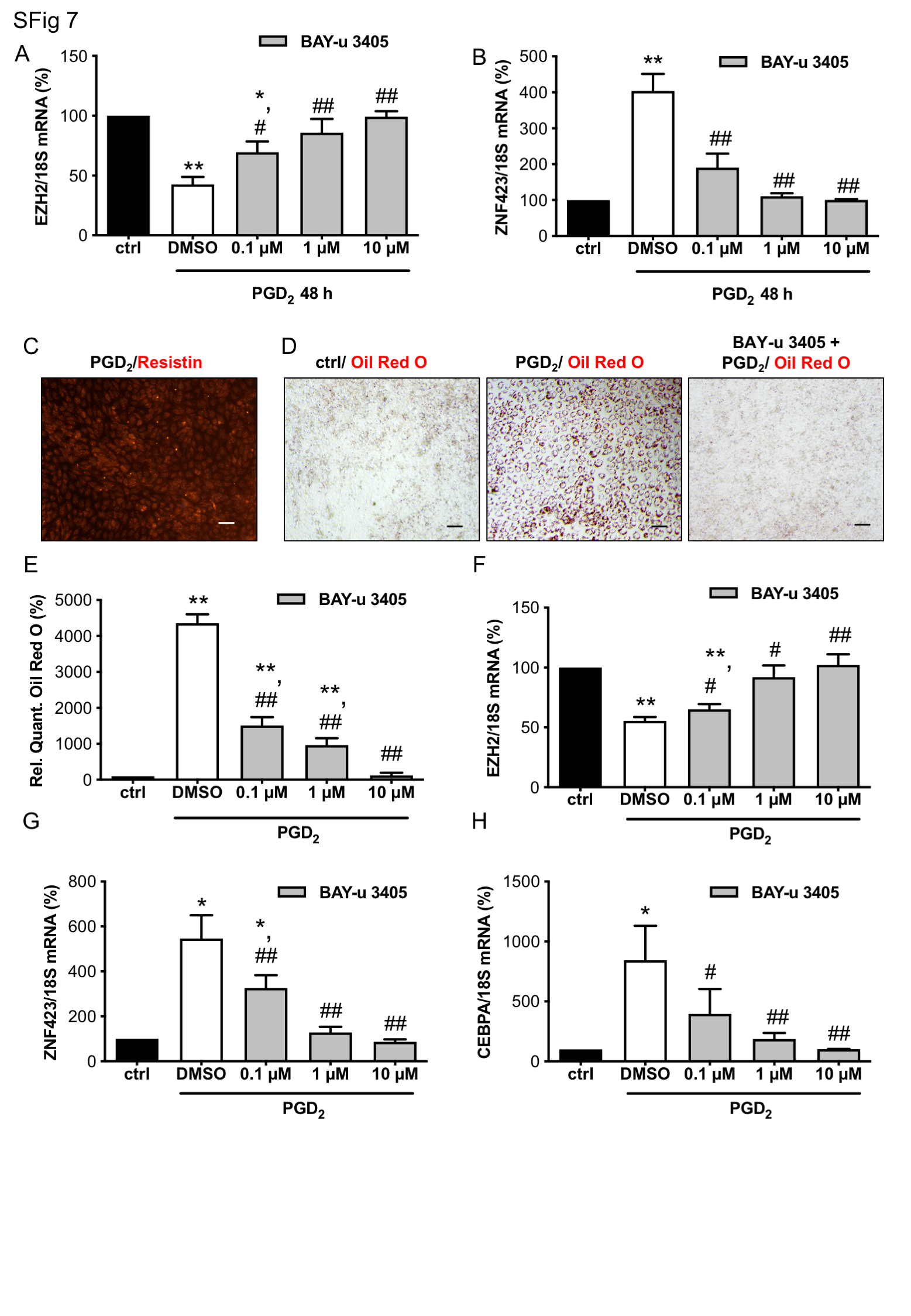

Supplement: S7 Fig — (A and B), Bar graphs summarizes (A) EZH2 and (B) ZNF423 mRNA expression of human iPSC 48 h after PGD2 stimulation (1 μM) and treatment with the DP2 receptor antagonist BAY-u 3405 in indicated concentrations (100 nm, 1 μM and 10 μM) for 48 h. (C and D) Representative pictures after (C) resistin (red) staining and (D) Oil Red O staining of PGD2-treated human iPSC after 12 days; scale bars: 50 μm. (E) Bar graphs summarize the relative quantification of Oil Red O measured by absorbance at 492 nm. (F–H) Bar graphs summarize mRNA levels assessed by qRT-PCR of (F) EZH2, (G) ZNF423, and (H) CEBPA of PGD2 (1 μM) treated-human iPSC incubated with BAY-u 3405 in indicated concentrations and cultivated for 12 days. N = 3 (A, B, F–H) and n = 6 (E) independent experiments. (A, B, E–H) Bar graphs represent mean ± SD, and the mean of control cells was set to 100%, * p <0.05, ** p < 0.01 vs. ctrl, # p < 0.05, ## p < 0.01 vs. PGD2 and DMSO-treated cells and 1 sample ttest. Underlying data can be found in S1 Data. CEBPA, CCAAT/enhancer-binding protein alpha; DP, PGD2 receptor; EZH2, enhancer of zeste homolog 2; iPSC, induced pluripotent stem cell; PGD2, prostaglandin D2; qRT-PCR, quantitative real-time PCR. (TIF) [file pbio.3000739.s007.tif]

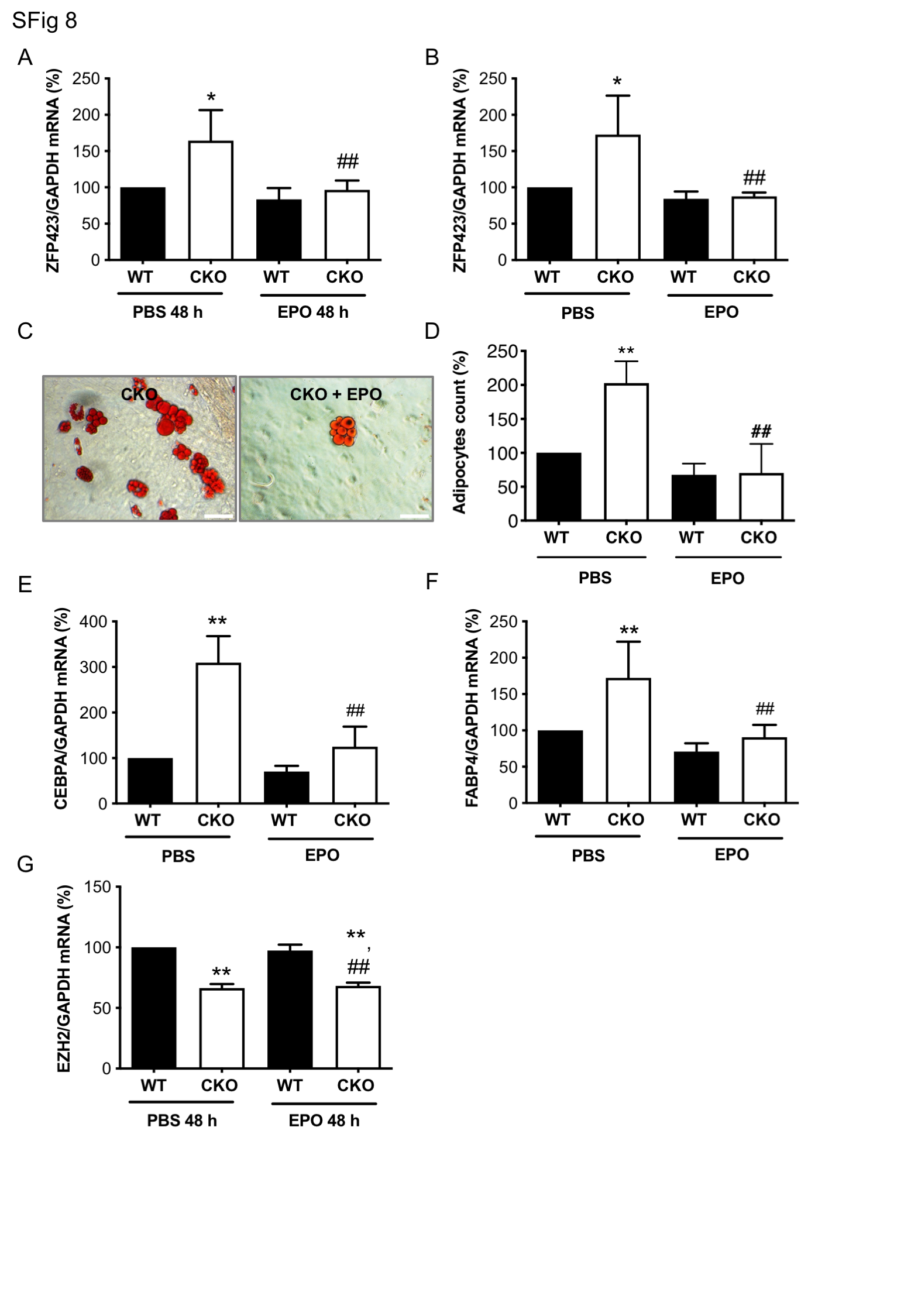

Supplement: S8 Fig — (A, B) Bar graphs summarize ZFP423 mRNA levels in isolated male CPC incubated with rmEPO (10 ng/ml) for (A) 48 h or (B) 4 weeks after isolation ((A): n = 3 cell isolations, each isolation consists of 8 to 12 mice per genotype and (B): n = 5 independent isolations, each isolation consists of 10 to 12 animals per genotype). (C) Oil Red O staining visualizes adipocytes in CKO-CPC cultures after 4 weeks of cultivation with or without the addition of rmEPO (10 ng/ml), scale bars: 50 μm. (D) Bar graph summarizing adipocyte counts (n = 5 independent isolations, each isolation consists of 10 to 12 animals per genotype). (E, F) qRT-PCR visualizes mRNA levels of the adipocyte markers (E) CEBPA and (F) FABP4 after 4 weeks of cultivation (n = 3 independent cell isolations, each isolation consists of 10 to 12 animals per genotype). (G) Bar graphs summarize EZH2 mRNA levels in isolated CPC incubated with rmEPO (10 ng/ml) for 48 h (n = 3 cell isolations, each isolation consists of 8 to 12 mice per genotype). (A, B, D–G) Data are presented as mean ± SD, and the mean of WT PBS was set at 100%, * p < 0.05, ** p < 0.01 vs. WT PBS, # p < 0.05, ## p < 0.01 vs. CKO PBS, 2-way ANOVA with Bonferroni's multiple comparison test. Underlying data can be found in S1 Data. CEBPA, CCAAT/enhancer-binding protein alpha; CKO, conditional knockout; CM, cardiomyocyte; CPC, cardiac progenitor cell; EPO, erythropoietin; EZH2, enhancer of zeste homolog 2; FABP4, fatty acid binding protein 4; qRT-PCR, quantitative real-time PCR; rmEPO, recombinant murine erythropoietin; WT, wild-type. (TIF) [file pbio.3000739.s008.tif]

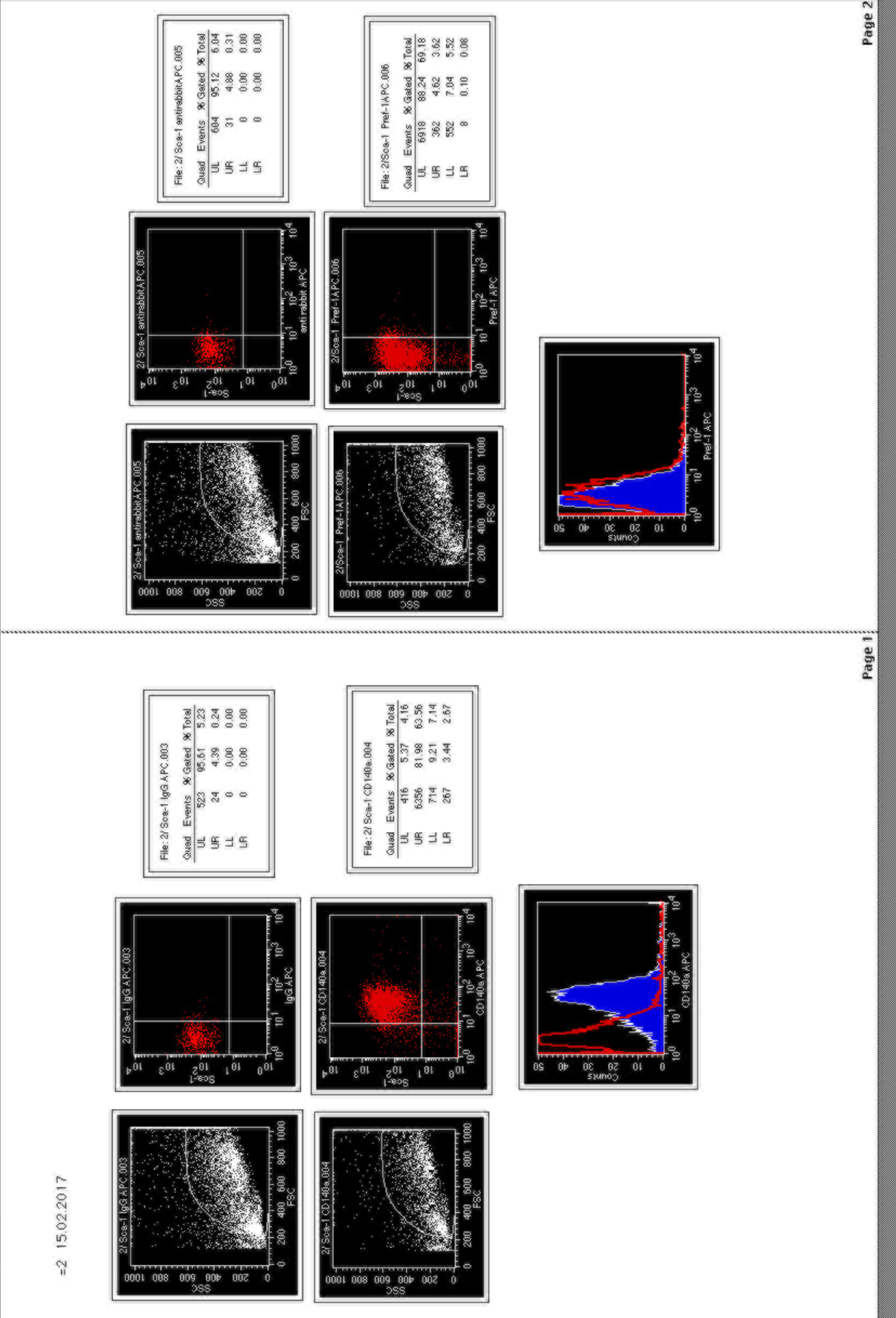

Supplement: S9 Fig — (TIF) [file pbio.3000739.s009.tif]

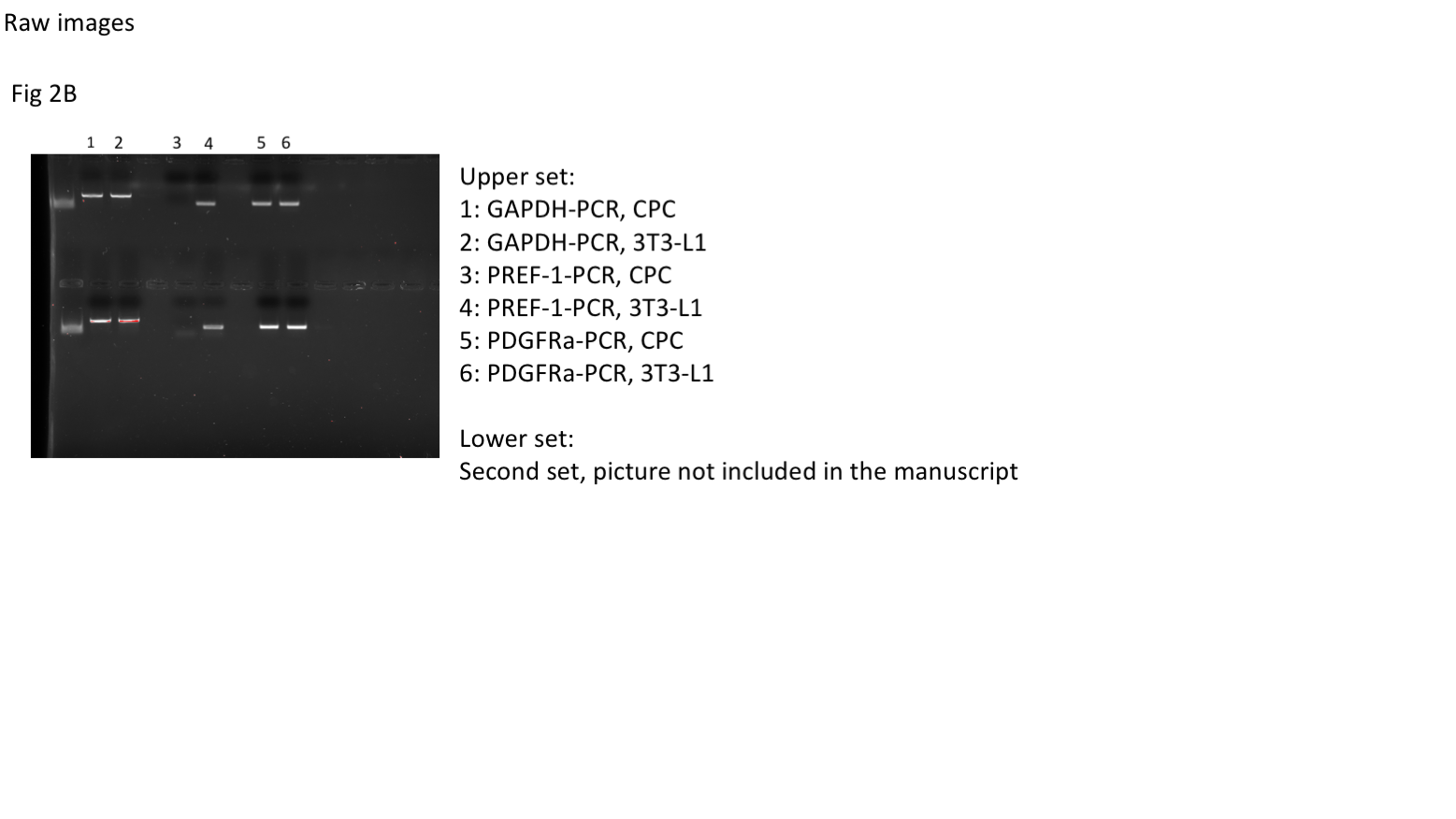

Supplement: S10 Fig — (TIFF) [file pbio.3000739.s010.tiff]

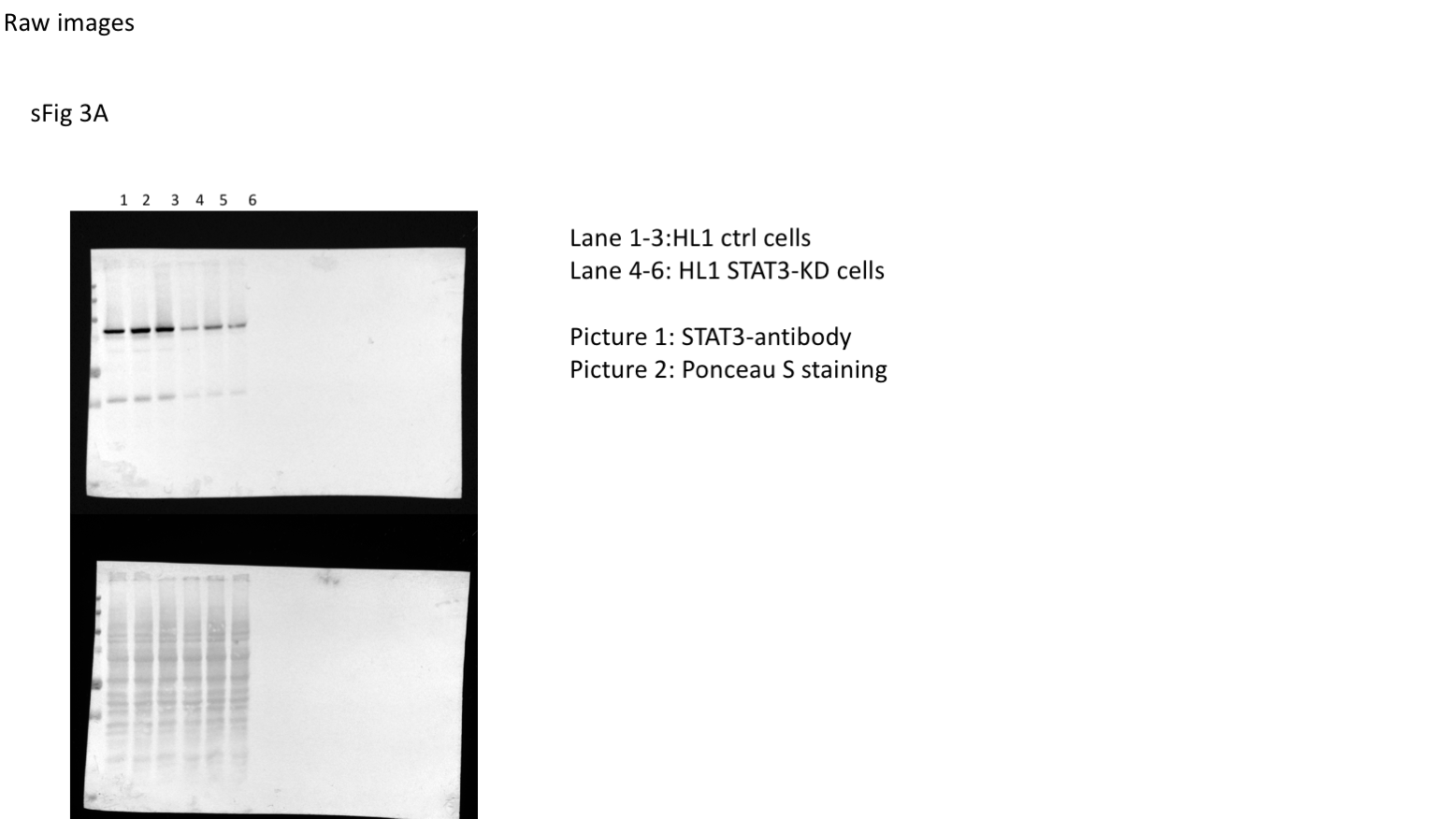

Supplement: S11 Fig — (TIFF) [file pbio.3000739.s011.tiff]
