# Supplementary material for: Increased prostaglandin-D2 in male STAT3-deficient hearts shifts cardiac progenitor cells from endothelial to white adipocyte differentiation
Source: PLoS Biol. 2020 Dec 28;18(12):e3000739. doi: 10.1371/journal.pbio.3000739 (PMC7793290; doi:10.1371/journal.pbio.3000739)
Supplement: S1 Table — FS, LVEDD, LVESD, and heart rate (bpm) in 3- and 6-month-old male mice. Data expressed as mean ± SD, * p <0.05, ** p <0.01 vs CKO 3 m, ## p < 0.01 vs WT 6 m, 2-way ANOVA, Bonferroni’s multiple comparison test. bpm, beats per minute; CKO, conditional knockout; FS, fractional shortening; LVEDD, left ventricular end-diastolic diameter; LVESD, left ventricular end-systolic diameter; WT, wild-type. (DOCX) [file pbio.3000739.s020.docx]

**S1 Table. Cardiac function and dimensions in male 3- and 6-month-old WT and CKO mice.**

|  | WT, 3 m (n=7) | CKO, 3 m (n=9) | WT, 6 m (n=10) | CKO, 6 m (n=10) |
| --- | --- | --- | --- | --- |
| %FS | 43±4 | 43±6 | 42±4 | 22±4**^,##^ |
| LVEDD (mm) | 3.9±0.4 | 3.6±0.2 | 4.0±0.4 | 4.2±0.4** |
| LVESD (mm) | 2.2±0.3 | 2.1±0.2 | 2.3±0.4 | 3.2±0.5**^,##^ |
| HR (bpm) | 482±29 | 497±50 | 515±34 | 521±42 |
