# Supplementary material for: Increased prostaglandin-D2 in male STAT3-deficient hearts shifts cardiac progenitor cells from endothelial to white adipocyte differentiation
Source: PLoS Biol. 2020 Dec 28;18(12):e3000739. doi: 10.1371/journal.pbio.3000739 (PMC7793290; doi:10.1371/journal.pbio.3000739)
Supplement: S2 Table — FS, LVEDD, LVESD, and heart rate (bpm) in 3- and 6-month-old female mice. Data expressed as mean ± SD, n.s., 2-way ANOVA, Bonferroni’s multiple comparison test. bpm, beats per minute; CKO, conditional knockout; FS, fractional shortening; LVEDD, left ventricular end-diastolic diameter; LVESD, left ventricular end-systolic diameter; WT, wild-type. (DOCX) [file pbio.3000739.s021.docx]

**S2 Table. Cardiac function and dimensions in female 3- and 6-month-old WT and CKO mice.**

|  | WT, 3 m (n=10) | CKO, 3 m (n=8) | WT, 6 m (n=9) | CKO, 6 m (n=7) |
| --- | --- | --- | --- | --- |
| %FS | 45±4 | 41±6 | 41±3 | 40±5 |
| LVEDD (mm) | 3.4±0.2 | 3.3±0.3 | 3.4±0.2 | 3.4±0.4 |
| LVESD (mm) | 1.8±0.1 | 1.9±0.2 | 2.0±0.2 | 3.8±0.5 |
| HR (bpm) | 544±29 | 526±93 | 498±39 | 484±31 |
