# Supplementary material for: Increased prostaglandin-D2 in male STAT3-deficient hearts shifts cardiac progenitor cells from endothelial to white adipocyte differentiation
Source: PLoS Biol. 2020 Dec 28;18(12):e3000739. doi: 10.1371/journal.pbio.3000739 (PMC7793290; doi:10.1371/journal.pbio.3000739)
Supplement: S3 Table — NYHA, LVEF, BP, and NT-proBNP were analyzed in routine clinical lab tests. Gaussian distribution was tested by D’Agostino–Pearson omnibus normality test Comparison between the groups was performed using Student t test for Gaussian distributed data (presented as mean ± SD) and the Mann Whitney U test where at least 1 column was not normally distributed (presented as median and range). BP, blood pressure; DCM, dilated cardiomyopathy; LVEF, left ventricular ejection fraction; NT-proBNP, N-terminal pro-brain natriuretic peptide; NYHA, New York Heart Association. (DOCX) [file pbio.3000739.s022.docx]

**S3 Table. Summary of clinical data from DCM patients and healthy sex-matched controls**

| Parameters | DCM  Males  (N=24) | Males  ctrl  (N=29) | DCM Females  (N=15) | Females  ctrl  (N=31) |
| --- | --- | --- | --- | --- |
| Age (years, mean ± SD) | 61±11 | 69±6 | 62±12 | 68±6 |
| NYHA: Median (range) | 2 (1-3) |  | 2 (2-3) |  |
| LVEF (%, mean ± SD) | 35±13 |  | 45±16 |  |
| Systolic BP (mmHg, mean ± SD) | 119±16 |  | 116±18 |  |
| Diastolic BP (mmHg, mean ± SD) | 77±8 |  | 75±9 |  |
| NT-proBNP (pmol/ml) Median (range) | 752 (57-63018) |  | 607 (49-2365) |  |
