# Supplementary material for: Increased prostaglandin-D2 in male STAT3-deficient hearts shifts cardiac progenitor cells from endothelial to white adipocyte differentiation
Source: PLoS Biol. 2020 Dec 28;18(12):e3000739. doi: 10.1371/journal.pbio.3000739 (PMC7793290; doi:10.1371/journal.pbio.3000739)
Supplement: S1 Text — (DOCX) [file pbio.3000739.s023.docx]

**Supplemental methods**

**Isolation, characterization and culture of SCA-1^+^ cardiac progenitor cells**

Isolation of SCA-1^+^ cells from hearts of 3-month-old mice was performed as described previously (1). In brief, after the mice were sacrificed, hearts were excised and washed with Hanks balanced salt solution (HBSS) (Merck). Aortic and pulmonary vessels and atria were removed. Tissues were minced into small pieces and digested for 30 min at 37 °C with 0.1 % collagenase type II (Worthington) in HBSS. The digested suspension was filtered through a 70 μm filter. Enrichment of SCA-1^+^ cells was achieved by sorting the cells using the SCA-1-FITC microbead kit (Miltenyi Biotec) and the Magnetic Cell Sorting (MACS) system (Miltenyi Biotec). Cells were purified by 4 cycles of magnetic sorting. Freshly isolated Sca-1^+^ cells were pre-plated for 2 h in DMEM/F12 (Merck) with 5 % FBS, and then seeded into plates pre-coated with fibronectin (10 μg/ml, Roche Applied Science) at a density of 11.000 cells/cm^2^. After 24 h, the medium was replaced by DMEM/F12 supplemented with ITS (10 μg/ml insulin, 5 μg/ml transferrin, 5 ng/ml sodium selenite) (Thermo Fisher Scientific), rhFGF-basic (10 ng/ml, R&D Systems) and epidermal growth factor (EGF; 20 ng/ml) for 6 days and subsequently switched to DMEM/F12 with 10 % FCS supplemented with ITS, rhFGF-basic and EGF. The medium was replaced every 2-3 days. Cells were maintained in culture under a humidified 5 % CO_2_ atmosphere at 37 °C for up to 4 weeks. rmEPO (10 ng/ml) was added to CPC cultures 24 h after seeding and replaced every third day. Controls were supplemented with the same volume of PBS. Alternatively, SCA-1^+^ cells were isolated in parallel with murine adult primary cardiomyocytes. Cardiomyocyte-depleted supernatants were subjected to magnetic cell sorting as described above.

**SCA-1^+^ cell cloning**

SCA-1^+^ cell cloning has been described previously (1, 2). In brief, 50-100 cells were spread on 100 mm dish containing SCA-1 growth medium. After one week, some of the isolated cells started growing and gave rise to distinct colonies after 3 weeks in culture. Colonies were marked (**∼**500 cells) for isolation and washed with HBSS. Sterile paper cloning discs were dipped in trypsin solution, placed on the top of the marked colonies and incubated for complete trypsinization. Cells were transferred quickly to a 24-well plate containing growth medium. After the cells approached confluence, they were expanded and characterized (2).

**Reduced Representation Bisulfite Sequencing (RRBS)**

**Table A. DNA concentration, amounts and qualities used for Bisulfite library preparation.**

| **Name** | **ID** | **DIN** | **ng/µl** | **frag. size (bp)** | **used DNA (ng)** |
| --- | --- | --- | --- | --- | --- |
| Set1 WT | 20180905_386_SH | 7.4 | 19.9 | 29729 | 100.0 |
| Set1 CKO | 20180905_386_SH | 7.5 | 54.1 | 51410 | 100.0 |
| Set2 WT | 20180905_386_SH | 7 | 46.7 | 51979 | 100.0 |
| Set2 CKO | 20180905_386_SH | 7.5 | 11.1 | 39437 | 94.4 |
| Set3 WT | 20180905_386_SH | 7.9 | 13.4 | 39437 | 100.0 |
| Set3 CKO | 20180905_386_SH | 7.9 | 22.5 | 49673 | 100.0 |

**Oil Red O staining**

For Oil Red O staining, CPC or 3T3-L1 cells were washed with PBS and fixed with 4 % paraformaldehyde for 15 min at 4 °C. Cells were washed twice with PBS and then washed for 10 min with 85 % propandiol at room temperature. Next, cells were stained for 1 h with Oil Red O solution (Sigma-Aldrich) and then washed twice for 10 min with 85 % propandiol and twice with PBS at room temperature. Cells were covered with Dako Mounting Medium (Dako). Alternatively, Oil Red O staining was quantified. For quantification cells were washed 3 times with 60 % isopropanol for 5 min each, Oil Red O stain was extracted using 100 % isopropanol for 5 min and absorbance was measured at 492 nm.

Adipocyte density was determined after Oil Red O staining on LV cryosections counterstained with hematoxylin. Cryosections mixed with cold 10% buffered formalin for 10 min were washed in tap water for 10 min and rinsed in distilled water for 5 min at room temperature. Slides were incubated for 2 min in 100 % propylene glycol to remove the water. Subsequently, sections were stained with freshly filtered Oil Red O solution for 80 min at 60 °C and were rinsed in distilled water for 5 min at room temperature. Cryosections were counterstained with hematoxylin for 15 s and washed in tap water for 5 min. Finally, slides were mounted in Dako mounting medium (Dako).

**Generation of human iPSC and stimulation with PGD_2_**

Human iPSC were generated as described previously (3), in brief CD34 peripheral human blood derived cells were reprogrammed using the Cytotune®-iPS 2.0 Sendai Reprogramming Kit according to the manufacturer's instructions. Single emerging hiPSC colonies were manually picked and transferred separately onto confluent layers of mitotically inactivated murine embryonic fibroblasts (miMEFs) in hiPSC medium consisting of Knockout DMEM supplemented with 20% Knockout Serum Replacement, 1% non-essential amino acid stock, 1 mM L-glutamine, 100 μM β-mercaptoethanol (all Thermo Scientific), and 10 ng/mL bFGF (supplied by the Institute for Technical Chemistry, Leibniz University Hannover, Germany). Cultures were split every 4–5 days using Collagenase VI (Thermo Scientific), plated at appropriate density onto fresh miMEFs and maintained at 37 °C and 5% CO2 (3)**.**

Human iPSC were grown in mTeSR^TM^1 (STEMCELL Technologies) on Matrigel^TM^ matrix (1:60 dilution, Corning) and passaged every 3-5 days using 1 mg/ml Dispase (STEMCELL Technologies). For adipocyte differentiation, cells were incubated with 0.5 mM EDTA for 5 min and 3x10^5^ cells were seeded per 12-well on Matrigel^TM^ matrix (1:60 dilution) in mTeSR^TM^1. The medium was changed every day until cells were confluent. Adipocyte differentiation was induced in DMEM/F12 supplemented with 10 % KnockOut^TM^ Serum Replacement (Thermo Fisher Scientific), 1 µM dexamethasone (G-Biosciences), 0.5 mM methylisobutylxanthine, 4 µg/ml human insulin and 1 µM prostaglandin D_2_ (Sigma Aldrich) for 48 h. Subsequently, medium was changed to DMEM/F12 supplemented with 10 % KnockOut^TM^ Serum Replacement, 4 µg/ml human insulin and 1 µM prostaglandin D_2_ for three weeks. The cells were harvested in TRIzol (Thermo Fisher Scientific) for RNA isolation or were fixed with 4 % paraformaldehyde.

**Stimulation of STAT3-deficient HL-1 cells**

In the cardiomyogenic HL-1 cells, the expression of STAT3 was silenced by a lentiviral transduction with anti-c-STAT3 shRNAs corresponding to position 823-841 of the murine c-*Stat3* gene (Genbank ID: NM_213659) (4). Lentiviral transgene plasmids pdc-SR and shRNAs served as controls. HL-1 cells were cultured at 37°C in a humidified atmosphere of 5 % CO_2_ (4, 5). HL-1 cells were stimulated with testosterone (10 nM, Serva) and estrogen (10 nM, Sigma-Aldrich). After 24 h cells were harvested in TRIzol.

**Table B. List of murine qRT-PCR primers.**

| **Transcript** | **Primer sequence** |
| --- | --- |
| ADGRE1 | For: GAGACATCCACTCTGGGCAC |
|  | Rev: GGGGCCCCTGTAGATACTGA |
|  |  |
| BMP2 | For: CTCTCTCAATGGACGTGCCC |
|  | Rev: AACACTAGAAGACAGCGGGTC |
|  |  |
| BMP4 | For: GCAACCCAGCCTGAGTATCT |
|  | Rev: ATGGCACTACGGAATGGCTC |
|  |  |
| CEBPA | For: TTCGGGTCGCTGGATCTCTA |
|  | Rev: TCAAGGAGAAACCACCACGG |
|  |  |
| COL1A1 | For: ACAGACGAACAACCCAAACT |
|  | Rev: GGTTTTTGGTCACGTTCAGT |
|  |  |
| COX-2 | For: TGCATTCTTTGCCCAGCACT |
|  | Rev: TCAGAGGCAATGCGGTTCTG |
|  |  |
| EBF2 | For: AGAGCAAGAAGGCTTGACCC |
|  | Rev: TGGCATGAGGGGTTATGAGC |
|  |  |
| EZH2 | For: AGCACAAGTCATCCCGTTAAAG |
|  | Rev: AATTCTGTTGTAAGGGCGACC |
|  |  |
| FABP44 | For: GTAAATGGGGATTTGGTCAC |
|  | Rev: GACTTTCCATCCCACTTCTG |
|  |  |
| GAPDH | For: ACCACCATGGAGAAGGCTGG |
|  | Rev: CTCAGTGTAGCCCAGGATGC |
|  |  |
| HPGD | For: CAACAATGCAGGCGTGAACA |
|  | Rev: GCGTGTGAATCCGATGATGC |
|  |  |
| LYZ2 | For: GAATGGAATGGCTGGCTACT |
|  | Rev: CGTGCTGAGCTAAACACACC |
|  |  |
| αMHC | For: GGAAGAGCGAGCGGCGCATCA AGG |
|  | Rev: GTCTGCTGGAGAGGTTATTCCTCG |
|  |  |
| PDGFRα | For: GACTGGAAGCTTGGGGCTTA |
|  | Rev: CTTCACTCTCCCCAACGCAT |
|  |  |
| PPARG2 | For: GCTGTTATGGGTGAAACTCTC |
|  | Rev: ATAAGCTGGAGATGCAGGTC |
|  |  |
| PRDM16 | For: GAGAGAGATTCCGCGAGCCG |
|  | Rev: CAGGATGCCGTCTTCGGTCT |
|  |  |
| PREF-1 | For: TGCGCGAACAATGGAACTTG |
|  | Rev: GCGGCTACGATCTCACAGAA |
|  |  |
| Resistin | For: AAGAACCTTTCATTTCCCCTCCT |
|  | Rev: GTCCAGCAATTTAAGCCAATGTT |
|  |  |
| SCA-1 | For: CTGTGCCTGCAACCTTGTCT |
|  | Rev: CACTGGTAACACTCCAGTCCC |
|  |  |
| TMEM26 | For: AGGGGCTTCCTTAGGGTTTTC |
|  | Rev: CCGTCTTGGATGAAGAAGCTG |
|  |  |
| UCP1 | For: CGTCCCCTGCCATTTACTGT |
|  | Rev: GACCCGAGTCGCAGAAAAGA |
|  |  |
| VE-Cadherin | For: CGTGGTGGAAACACAAGATG |
|  | Rev: CGTTTGGGTCTGTCTCAAT |
|  |  |
| ZFP423 | For: CAGGCCCACAAGAAGAACAAG |
|  | Rev: GTATCCTCGCAGTAGTCGCACA |
|  |  |
| ZFP521 | For: TTACGTACAGAGCGAGTCCC |
|  | Rev: GAGGGATCTCGGTTTCGCTT |
|  |  |
| 18S | For: GTAACCCGTTGAACCCCATT |
|  | Rev: CCATCCAATCGGTAGTAGCG |

**Table C. List of human qRT-PCR primers.**

| **Transcript** | **Primer sequence** |
| --- | --- |
| CEBPA | For: TATAGGCTGGGCTTCCCCTT |
|  | Rev: AGCTTTCTGGTGTGACTCGG |
|  |  |
| COX-2 | For: GATGATTGCCCGACTCCCTT |
|  | Rev: TGAAAAGGCGCAGTTTACGC |
|  |  |
| EZH2 | For: CCCTGACCTCTGTGTTACTTGTGGA |
|  | Rev: ACGTCAGATGGTGCCAGCAATA |
|  |  |
| HPGD | For: CATGCACGTGAACGGCAAA |
|  | Rev: CGGGCATGAGTCCTGCTAAA |
|  |  |
| 18S | For: AGAACGAAAGTCGGAGGTTCG |
|  | Rev: GGACATCTAAGGGCATCACAG |

**References**

1. Hoch M, Fischer P, Stapel B, Missol-Kolka E, Sekkali B, Scherr M, et al. Erythropoietin preserves the endothelial differentiation capacity of cardiac progenitor cells and reduces heart failure during anticancer therapies. Cell stem cell. 2011;9(2):131-43.

2. De Pauw A, Massion P, Sekkali B, Andre E, Dubroca C, Kmecova J, et al. Paracrine nitric oxide induces expression of cardiac sarcomeric proteins in adult progenitor cells through soluble guanylyl cyclase/cyclic-guanosine monophosphate and Wnt/beta-catenin inhibition. Cardiovascular research. 2016;112(1):478-90.

3. Pongpamorn P, Dahlmann J, Haase A, Ebeling CT, Merkert S, Gohring G, et al. Generation of three induced pluripotent stem cell lines (MHHi012-A, MHHi013-A, MHHi014-A) from a family with Loeys-Dietz syndrome carrying a heterozygous p.M253I (c.759G>A) mutation in the TGFBR1 gene. Stem Cell Res. 2020;43:101707.

4. Elschami M, Scherr M, Philippens B, Gerardy-Schahn R. Reduction of STAT3 expression induces mitochondrial dysfunction and autophagy in cardiac HL-1 cells. European journal of cell biology. 2013;92(1):21-9.

5. White SM, Constantin PE, Claycomb WC. Cardiac physiology at the cellular level: use of cultured HL-1 cardiomyocytes for studies of cardiac muscle cell structure and function. American journal of physiology Heart and circulatory physiology. 2004;286(3):H823-9.
