# Supplementary material for: Increased prostaglandin-D2 in male STAT3-deficient hearts shifts cardiac progenitor cells from endothelial to white adipocyte differentiation
Source: PLoS Biol. 2020 Dec 28;18(12):e3000739. doi: 10.1371/journal.pbio.3000739 (PMC7793290; doi:10.1371/journal.pbio.3000739)
Supplement: Attachment — Submitted filename: Rebuttal PLoS Biologyfinal.docx [file pbio.3000739.s024.docx]

**Editors comments**

1. **Importantly, we would like to see evidence that the PGD2-Zfp423 axis is a critical pathway driving adipogenesis in vivo (reviewer 2):**

Dear Editor, as requested we addressed this important question in the revised manuscript by blocking the PGD2 generating enzyme COX2 by treating CKO mice with the COX inhibitor Ibuprofen for two weeks to investigate whether this would reduce the adipocyte priming of cardiac progenitor cells. Indeed, isolated CKO-CM from Ibuprofen-treated CKO mice released less PGD2 and CKO-CPC from CKO mice treated with Ibuprofen displayed lower expression of ZFP423 and higher expression of EZH2 compared to CPC from not treated CKO mice. Moreover, cultivation of CPC from Ibuprofen treated CKO mice displayed reduced adipocyte differentiation compared to CPC from not treated CKO mice.

However, in order to analyze whether Ibuprofen treatment would be beneficial to reduce age-related heart failure in CKO mice, we would have to treat mice for at least 3-4 months. Since long-term use of nonsteroidal anti-inflammatory drugs like Ibuprofen has been described to generate gastrointestinal problems in mice such long-term treatment would generate unpredictable side effects in mice and we therefore did not do this experiment.

The additional data on Ibuprofen treatment were added to the revised manuscript:

**Result section, page 12, line 285:**

*“Treatment of young male CKO mice with the COX-inhibitor Ibuprofen or the DP2 receptor antagonist BAY-u 3405 reduced adipocyte differentiation of CKO-CPC*

*In order to evaluate whether PGD_2_ is responsible for the enhanced adipocyte differentiation potential of CPC in CKO male mice, young male CKO mice before heart failure onset were treated either with the COX-inhibitor Ibuprofen or the DP2 antagonist BAY-u 3405 in vivo. After two weeks treatment, PGD_2_ levels were reduced in the supernatants of isolated CKO-CM of Ibuprofen but not BAY-u 3405 treated CKO mice compared to levels in the supernatants of CKO-CM isolated from control CKO mice (Figs 6A and 6B). CKO-CPC isolated from Ibuprofen- or BAY-u 3405-treated CKO mice displayed enhanced EZH2 expression and reduced ZFP423 expression compared to control CKO-CPC (Figs 6C-6F). The spontaneous adipocyte differentiation of CKO-CPC isolated from Ibuprofen or BAY-u 3405 treated CKO mice was abolished compared to CKO-CPC isolated from untreated CKO mice (Figs 6G and 6H).”*

**Discussion section: Page 14, line 319:**

*“PGD_2_ is generated by multiple enzymatic steps from arachidonic acid and COX-2 is a rate limiting enzyme in this process (34). COX-2 is elevated in CM from female and male CKO mice, an observation that contrasts with findings showing that STAT3 is a direct transcription factor of COX-2 in ischemic preconditioning, where activation of cardiac STAT3 upregulates COX-2 in the heart (35). In fact, beneficial effects of COX-2 in protection of the heart from ischemic injury have been reported also indicating that the use of COX inhibitors may have adverse effects in the setting of ischemia/reperfusion (I/R) (36)). In contrast, protective effects of COX-2 inhibition on cardiomyocytes have also been reported (37). The present study analyses pathomechanisms of age-related idiopathic or dilated cardiomyopathy (DCM) for which pathomechanism are different from myocardial infarction or I/R injury as for example canonical STAT3 signaling is not or only moderately activated. Our data suggest that inactive STAT3 acts either directly or indirectly as a negative regulator of COX-2, a feature that will be explored in future studies.”*

**Page 15, line 342:**

*“This deregulated arachidonic metabolism results in enhanced PGD_2_ production in cardiomyocytes which could also be confirmed in vivo as the treatment of CKO male mice with the COX-inhibitor Ibuprofen lowered the PGD_2_ secretion in CKO-CM. Thus, we provide evidence that enhanced COX-2 and sex-specific reduced HPGD contributes to elevated PGD_2_ production in male failure prone and failing hearts with low STAT3 expression (Fig 7)”*

**Page 15, line 359:**

“The white adipocyte formation by PGD_2_ is mediated by the DP2-receptor since it could be inhibited by the DP2 receptor antagonist BAY-u 3405 but not by the DP1 receptor antagonist BWA868C. Moreover, since the treatment of CKO mice with the DP2 receptor antagonist BAY-u 3405 reduced the enhanced adipocyte formation of CKO-CPC, the role of PGD_2_ via its DP2 receptor for the formation of WAT could also be confirmed in vivo.”

**Page 18, line 430:**

*“*Finally, the present data suggest that the COX inhibitor Ibuprofen or the DP2 receptor antagonist BAY-u 3405 may attenuate these remodeling process and with this reduce age-related heart failure specifically in males with impaired AR signaling.

Limitation to this study: Although, we could show that COX inhibition and more specifically inhibition of the PGD_2_ receptor DP2 prevents the enhanced white adipocyte formation of CPC in failure prone CKO male hearts, we have not performed chronic treatment with COX inhibitors or BAY-u 3405 to test whether they would impact on age-related heart failure of the DCM phenotype.*”*

**Method section, page 19, line 455:**

*“Ibuprofen and BAY-u 3405 treatment of CKO mice*

*Male CKO mice in the age of 9-10 weeks were treated with Ibuprofen (10 mg/kg bodyweight, Ibuprofen sodium salt, Sigma-Aldrich, dissolved in drinking water) or BAY-u 3405 (10 mg/kg bodyweight, Ramatroban, medchemexpress, dissolved in drinking water) for two weeks. CKO control animals received drinking water.”*

2. **More in-depth analyses of the human tissue samples (reviewer 1, point 3).**

We addressed also this important question of reviewer 1. However, unfortunately, the material from human hearts, especially from non-failing female hearts, is limited so that we were not able to do further analyses with regard to sex-differences or adipocyte staining in human hearts. However, PGD_2_ is a secreted factor, we were able to measure serum content in female and male patients with heart failure due to dilated cardiomyopathy and age- and sex-matched healthy controls. We observed significantly higher levels of circulating PGD_2_ in male but not female patients in comparison to their respective controls. This new data (added to the revised manuscript as indicated below) together with our observation that PGD_2_ is inducing white adipocyte differentiation in human iPSC and the data from human hearts showing reduced HPGD expression and increased WAT marker expression strongly supports the notion that a similar mechanism is also present in male patients with DCM related heart failure. The data are added to the revised manuscript as indicated below:

**The data are presented in the revised result section, page 8, starting line 172:**

*“Serum levels of PGD_2_ are increased in male patients with heart failure but not in female patients with heart failure*

*Male patients with heart failure due to idiopathic dilated cardiomyopathy (DCM) showed elevated PGD_2_ serum levels compared to serum from healthy age-matched males whereas PGD_2_ serum levels from females with heart failure due to DCM showed no difference compared to serum from healthy age-matched females (Figs 1K and 1L, clinical data of this cohort are shown in Tab. S3).”*

**The discussion section, page 18, line 419:**

*“Moreover, male patients but not female patients with heart failure due to DCM displayed elevated PGD_2_ serum levels suggesting that sex-specific enhanced PGD_2_ are also present in humans with heart failure.”*

**The method section, page 26, line 642:**

*“The local ethics committee of Hannover Medical School approved this study. All patients provided written informed consent. The study conforms to the principles outlined in the declaration of Helsinki. All patients (males N=24, females N=15) enrolled in the study were diagnosed with heart failure due to idiopathic dilated cardiomyopathy (DCM). The sex- and age-matched control collective consisted of healthy males (N=29) and females (N=31) who displayed normal LVEF (>55%), blood pressure and heart rates.”*

**And page 26, line 653**

*Blood tests*

*“Blood samples were collected in S-Monovette® tubes containing ethylenediaminetetraacetic acid (EDTA, for plasma) or clot activator (for serum) in patients with the diagnosis for DCM or PPCM. Plasma or serum were separated by centrifugation at 1500 rpm for 10 min and aliquots were stored at -80 °C. Laboratory workup was performed as part of routine analysis by hospital laboratories. PGD_2_ serum levels were measured using the prostaglandin D_2_ ELISA kit (Cayman Chemicals No. 512031) according to the manufacturer’s protocols.”*

*****************************************************
REVIEWS:

**Reviewer #1: The study by Stelling et al. presents interesting results on how STAT3 deficiency drives left ventricular dysfunction during aging in male mice. The authors present clear evidence that a shift from differentiation of progenitor cells from endothelial cells towards adipocytes occurs in aged male STAT3 deficeint mice mainly driven by increased release of prostaglanin D2 due to increased activity of cyclooxygenase 2 and less activity of HPGD. The data are further supported by extensive in-vitro work on progenitor cells which,  most interestingly keep their phenotype even after long term cultivation. The experiments are well conducted and I have only a few points the authors might want to consider:**

**1.    The release of PGD2 from isolated cardiomyocytes was normalized to total RNA. Why did the authors choose total RNA rather than a cardiomyocyte specific protein (troponin, alphaMHC) to account for cardiomyocyte number? Total RNA could still be derived from contaminating other cells.**

We thank the reviewer for the comment and changed the normalization of the release of PGD_2_ from isolated cardiomyocytes to alpha-MHC (see Figs 1I and 1J and Figs 6A and 6B).

**2.    Did the authors verify the changes observed in the immunohistology (figure 1) on capillary density and fibrosis by other techniques (Western blot: CD31, collagen etc)? Both the changes in capillary density and fibrosis (which appears to be very small (1-3%)) are not very impressive and most likely does not account for heart failure development.**

We thank the reviewer for the comment.

Please note the aim of the present study was to analyze why male but not female CKO mice develop age-related heart failure and not the heart failure phenotype per se. In fact, the age-related heart failure phenotype including reduced capillary density and fibrosis of STAT3-KO male mice has been described previously including quantification of fibrosis and endothelial markers (Hilfiker-Kleiner et al. Circ Res 2004; Hoch et al. Cell Stem Cell 2011). We therefore moved the data on capillarization (presented as capillaries per cardiomyocyte), fibrosis and inflammation to the supplementary Figures (S1 Fig), where we also added VE-Cadherin and COL1A1 mRNA expression confirming these changes also on the molecular level.

**3.    The human data are interesting but very superficial. I am aware that obtaining human tissue might be complicated but at least the tissue available should be analyzed further: markers for endothelial cell content, adipocyte content etc. Even more, some data from female heart failure patients would be of interest to proof that these parameters might differ depending on sex.**

We thank the reviewer for his/her comment. Unfortunately, the material from human hearts, especially from non-failing female hearts, is limited so that we were not able to do further analyses with regard to sex-differences or adipocyte staining in human hearts. However, since PGD_2_ is a secreted factor, we were able to measure serum content in female and male patients with heart failure due to dilated cardiomyopathy and age- and sex-matched healthy controls showing a significant increase of circulating PGD_2_ in male but not female patients in comparison to their respective controls.

**The data are presented in the revised result section, page 8, starting line 172:**

*“Serum levels of PGD_2_ are increased in male patients with heart failure but not in female patients with heart failure*

*Male patients with heart failure due to idiopathic dilated cardiomyopathy (DCM) showed elevated PGD_2_ serum levels compared to serum from healthy age-matched males whereas PGD_2_ serum levels from females with heart failure due to DCM showed no difference compared to serum from healthy age-matched females (Figs 1K and 1L, clinical data of this cohort are shown in Tab. S3).”*

**The discussion section, page 18, line 419:**

*“Moreover, male patients but not female patients with heart failure due to DCM displayed elevated PGD_2_ serum levels suggesting that sex-specific enhanced PGD_2_ are also present in humans with heart failure.”*

**The method section, page 26, line 642:**

*“The local ethics committee of Hannover Medical School approved this study. All patients provided written informed consent. The study conforms to the principles outlined in the declaration of Helsinki. All patients (males N=24, females N=15) enrolled in the study were diagnosed with heart failure due to idiopathic dilated cardiomyopathy (DCM). The sex- and age-matched control collective consisted of healthy males (N=29) and females (N=31) who displayed normal LVEF (>55%), blood pressure and heart rates.”*

**And page 26, line 653**

*Blood tests*

*“Blood samples were collected in S-Monovette® tubes containing ethylenediaminetetraacetic acid (EDTA, for plasma) or clot activator (for serum) in patients with the diagnosis for DCM or PPCM. Plasma or serum were separated by centrifugation at 1500 rpm for 10 min and aliquots were stored at -80 °C. Laboratory workup was performed as part of routine analysis by hospital laboratories. PGD_2_ serum levels were measured using the prostaglandin D_2_ ELISA kit (Cayman Chemicals No. 512031) according to the manufacturer’s protocols.”*

**4.    The authors demonstrate that COX2 might be a key protein in age-dependent or post-partum triggered heart failure development in STAT3-deficiency. Does this mean that the phenotype can be rescued by COX2 inhibition? Did the authors try to treat aged male mice with COX2 inhibitors? In this context, the discussion on COX2 (PMID: 31734378; PMID: 31380437; PMID: 31278427 to name only a few recent studies) on ischemia-reperfusion related cardiac effects (which involves remodelling and heart failure development as well) needs to be extended.**

We thank the reviewer for raising this important point. We followed the reviewer’s suggestion and treated CKO mice with the COX inhibitor Ibuprofen for two weeks to investigate whether this would reduce the adipocyte priming of CKO-CPC. Indeed, isolated CKO-CPC from CKO mice treated with Ibuprofen displayed lower expression of ZFP423 and higher expression of EZH2 compared to CPC from not treated CKO mice. Moreover, cultivation of CPC from Ibuprofen treated CKO mice displayed reduced adipocyte differentiation compared to CPC from not treated CKO mice.

However, in order to analyze whether Ibuprofen treatment would be beneficial to reduce age-related heart failure in CKO mice, we would have to treat mice for at least 3-4 months. Since long-term use of nonsteroidal anti-inflammatory drugs like Ibuprofen has been described to generate gastrointestinal problems in mice such long-term treatment would generate unpredictable side effects in mice and we therefore did not do this experiment.

The additional data on Ibuprofen treatment were added to the revised manuscript:

**Result section, page 12, line 285:**

*“Treatment of young male CKO mice with the COX-inhibitor Ibuprofen or the DP2 receptor antagonist BAY-u 3405 reduced adipocyte differentiation of CKO-CPC*

*In order to evaluate whether PGD_2_ is responsible for the enhanced adipocyte differentiation potential of CPC in CKO male mice, young male CKO mice before heart failure onset were treated either with the COX-inhibitor Ibuprofen or the DP2 antagonist BAY-u 3405 in vivo. After two weeks treatment, PGD_2_ levels were reduced in the supernatants of isolated CKO-CM of Ibuprofen but not BAY-u 3405 treated CKO mice compared to levels in the supernatants of CKO-CM isolated from control CKO mice (Figs 6A and 6B). CKO-CPC isolated from Ibuprofen- or BAY-u 3405-treated CKO mice displayed enhanced EZH2 expression and reduced ZFP423 expression compared to control CKO-CPC (Figs 6C-6F). The spontaneous adipocyte differentiation of CKO-CPC isolated from Ibuprofen or BAY-u 3405 treated CKO mice was abolished compared to CKO-CPC isolated from untreated CKO mice (Figs 6G and 6H).”*

**Discussion section: Page 14, line 319:**

*“PGD_2_ is generated by multiple enzymatic steps from arachidonic acid and COX-2 is a rate limiting enzyme in this process (34). COX-2 is elevated in CM from female and male CKO mice, an observation that contrasts with findings showing that STAT3 is a direct transcription factor of COX-2 in ischemic preconditioning, where activation of cardiac STAT3 upregulates COX-2 in the heart (35). In fact, beneficial effects of COX-2 in protection of the heart from ischemic injury have been reported also indicating that the use of COX inhibitors may have adverse effects in the setting of ischemia/reperfusion (I/R) (36)). In contrast, protective effects of COX-2 inhibition on cardiomyocytes have also been reported (37). The present study analyses pathomechanisms of age-related idiopathic or dilated cardiomyopathy (DCM) for which pathomechanism are different from myocardial infarction or I/R injury as for example canonical STAT3 signaling is not or only moderately activated. Our data suggest that inactive STAT3 acts either directly or indirectly as a negative regulator of COX-2, a feature that will be explored in future studies.”*

**Page 15, line 342:**

*“This deregulated arachidonic metabolism results in enhanced PGD_2_ production in cardiomyocytes which could also be confirmed in vivo as the treatment of CKO male mice with the COX-inhibitor Ibuprofen lowered the PGD_2_ secretion in CKO-CM. Thus, we provide evidence that enhanced COX-2 and sex-specific reduced HPGD contributes to elevated PGD_2_ production in male failure prone and failing hearts with low STAT3 expression (Fig 7)”*

**Page 15, line 359:**

*“The white adipocyte formation by PGD_2_ is mediated by the DP2-receptor since it could be inhibited by the DP2 receptor antagonist BAY-u 3405 but not by the DP1 receptor antagonist BWA868C. Moreover, since the treatment of CKO mice with the DP2 receptor antagonist BAY-u 3405 reduced the enhanced adipocyte formation of CKO-CPC, the role of PGD_2_ via its DP2 receptor for the formation of WAT could also be confirmed in vivo.”*

**Page 18, line 430:**

*“Finally, the present data suggest that the COX inhibitor Ibuprofen or the DP2 receptor antagonist BAY-u 3405 may attenuate these remodeling process and with this reduce age-related heart failure specifically in males with impaired AR signaling.*

*Limitation to this study: Although, we could show that COX inhibition and more specifically inhibition of the PGD_2_ receptor DP2 prevents the enhanced white adipocyte formation of CPC in failure prone CKO male hearts, we have not performed chronic treatment with COX inhibitors or BAY-u 3405 to test whether they would impact on age-related heart failure of the DCM phenotype.”*

**Method section, page 19, line 455:**

*“Ibuprofen and BAY-u 3405 treatment of CKO mice*

*Male CKO mice in the age of 9-10 weeks were treated with Ibuprofen (10 mg/kg bodyweight, Ibuprofen sodium salt, Sigma-Aldrich, dissolved in drinking water) or BAY-u 3405 (10 mg/kg bodyweight, Ramatroban, medchemexpress, dissolved in drinking water) for two weeks. CKO control animals received drinking water.”*

**Reviewer #2: Reduced expression and activity of cardiac STAT3 is linked to dilatative cardiomyopathy or peripartum cardiomyopathy; however, the mechanisms underlying pathologic cardiac remodeling triggered upon cardiomyocyte STAT3 inactivation has remained unclear.  Here, the authors propose that cardiomyocyte-specific STAT3 deficiency leads increased PGD2 secretion from male cardiomyocyte but not from female cardiomyocytes.  Increased PGD2 in the microenvironment then leads to suppression of EZH2 in cardiac progenitor cells (defined here as Sca-1+ positive cells) and subsequent activation of Zfp423, a transcription factor driving adipocyte differentiation.  The authors propose that PGD2 driven adipogenesis from the Sca-1+ positive cells drive the ectopic adipocyte accumulation and loss of vascular density.

Overall, the manuscript is well written and the hypothesis is certainly novel and of general interest.   The major concern lies in whether the states hypothesis is sufficiently supported by the data on hand.  The authors demonstrate convincingly in vitro that PGD2 can activate Zfp423 expression in progenitor cells and promote adipogenesis.**

1) **What is missing is evidence that this is the critical pathway driving adipogenesis in vivo in this model.  In fact, any number of secreted factors from STAT3-deficient cardiomyocytes might influence adipogenesis.   As such, the current observations establish associations, but not a clear sense of causality of the phenotype in vivo. For example, does inactivation of PGD2 receptor in Sca-1+ cells rescue the phenotype?**

We thank the reviewer for this suggestion. We treated cCPC cells with PGD_2_ with and without the PGD_2_ receptor DP2 and DP1 antagonists and could show that the PGD_2_-induced increased adipocyte differentiation of CPC was abrogated by the DP2 antagonist BAY-u 3405 (Ramatroban) but not by DP1 receptor antagonist BWA868C. BAY-u 3405 abolished also PGD_2_-induced adipocyte differentiation in human iPSC. The new data are added to the revised manuscript:

**Result section: Page 12, line 267:**

*“PGD_2-_induced adipocyte differentiation in clonally expanded CPC that could be prevented by the PGD_2_ receptor 2 antagonist BAY-u 3405 but not by the PGD_2_ receptor 1 antagonist BWA868C*

*Treatment of clonally expanded CPC (cCPC) with PGD_2_ induced adipocyte differentiation (Figs 5A and 5H). The PGD_2_-induced adipocyte differentiation in cCPC could be attenuated by the PGD_2_ receptor 2 (DP2) antagonist BAY-u 3405 while incubation with the DP1 receptor antagonist BWA868C did not influence PGD_2_ induced ZFP423 expression or adipocyte differentiation (Figs 5A-5H).”*

*PGD_2_ induced adipocyte differentiation from human iPSC*

*PGD_2_ treatment for 48 h reduced EZH2 expression and increased ZNF423 expression in human induced pluripotent stem cells (iPSC) (S7A and S7B Figs). Prolonged treatment with PGD_2_ induced also white adipocyte differentiation of iPSC (Resistin and Oil Red O staining, S7C and S7D Figs). In addition, ZNF423 and CEBPA expression were elevated and EZH2 expression was reduced in PGD_2_ treated human iPSC compared to control iPSC (S7F-S7H Figs). All aspects of adipocyte differentiation induced by PGD_2_ in iPSC could be attenuated by BAY-u 3405 (Figs S7E-S7H)”.*

**Discussion section:**

**Page 14, line 312**

*“The shift of CPC towards adipocyte differentiation could be attenuated by treatment of CKO mice with the COX inhibitor Ibuprofen or the DP2 receptor antagonist BAY-u 3405 thereby confirming the role of PGD_2_ for cardiac WAT generation in vivo.”*

**Page 15, line 359**

*“The white adipocyte formation by PGD_2_ is mediated by the DP2-receptor since it could be inhibited by the DP2 receptor antagonist BAY-u 3405 but not by the DP1 receptor antagonist BWA868C. Moreover, since the treatment of CKO mice with the DP2 receptor antagonist BAY-u 3405 reduced the enhanced adipocyte formation of CKO-CPC, the role of PGD_2_ via its DP2 receptor for the formation of WAT could also be confirmed in vivo.”*

**Page 18, line 430:**

“Finally, the present data suggest that the COX inhibitor Ibuprofen or the DP2 receptor antagonist BAY-u 3405 may attenuate these remodeling process and with this reduce age-related heart failure specifically in males with impaired AR signaling.”

**Method section: Page 24, line 581:**

*“Treatment of cCPC and human iPSC with DP receptor antagonists*

*Clonally expanded CPC and human iPSC were incubated with the DP1 receptor antagonist BWA868C and the DP2 receptor antagonist BAY-u 3405 (1 µM, 10 µM in DMSO, both from Cayman Chemicals) starting 2 h prior PGD_2_ treatment. In addition, cells were treated with PGD_2_ and DMSO. Cells were cultivated for 48 h or for two weeks.”*

**2. Further evidence that this pathway is mediating the phenotype in vivo would strengthen the conclusions.**

As mentioned above, we first identified the responsible PGD_2_ receptor for PGD_2_-induced adipocyte differentiation of CPC and showed that it is the DP2. Next, we treated CKO mice with the DP2 antagonist BAY-u 3405 for two weeks and observed that CPCs from BAY-u 3405 treated CKO mice displayed significantly lower levels of ZFP423 and increased expression of EZH2. In addition, cultivation of CPC from BAY-u 3405 treated CKO mice displayed less adipocyte formation compared to CPC from not treated CKO mice.

The additional data are included in the manuscript and are in part already shown in the previous answer. Here are the additional parts with regard to the *in vivo* treatment

**Result section, page 12, line 285:**

*“Treatment of young male CKO mice with the COX-inhibitor Ibuprofen or the DP2 receptor antagonist BAY-u 3405 reduced adipocyte differentiation of CKO-CPC*

*In order to evaluate whether PGD_2_ is responsible for the enhanced adipocyte differentiation potential of CPC in CKO male mice, young male CKO mice before heart failure onset were treated either with the COX-inhibitor Ibuprofen or the DP2 antagonist BAY-u 3405 in vivo. After two weeks treatment, PGD_2_ levels were reduced in the supernatants of isolated CKO-CM of Ibuprofen but not BAY-u 3405 treated CKO mice compared to levels in the supernatants of CKO-CM isolated from control CKO mice (Figs 6A and 6B). CKO-CPC isolated from Ibuprofen- or BAY-u 3405-treated CKO mice displayed enhanced EZH2 expression and reduced ZFP423 expression compared to control CKO-CPC (Figs 6C-6F). The spontaneous adipocyte differentiation of CKO-CPC isolated from Ibuprofen or BAY-u 3405 treated CKO mice was abolished compared to CKO-CPC isolated from untreated CKO mice (Figs 6G and 6H).”*

**Discussion section: Page 14, line 319:**

*“PGD_2_ is generated by multiple enzymatic steps from arachidonic acid and COX-2 is a rate limiting enzyme in this process (34). COX-2 is elevated in CM from female and male CKO mice, an observation that contrasts with findings showing that STAT3 is a direct transcription factor of COX-2 in ischemic preconditioning, where activation of cardiac STAT3 upregulates COX-2 in the heart (35). In fact, beneficial effects of COX-2 in protection of the heart from ischemic injury have been reported also indicating that the use of COX inhibitors may have adverse effects in the setting of ischemia/reperfusion (I/R) (36)). In contrast, protective effects of COX-2 inhibition on cardiomyocytes have also been reported (37). The present study analyses pathomechanisms of age-related idiopathic or dilated cardiomyopathy (DCM) for which pathomechanism are different from myocardial infarction or I/R injury as for example canonical STAT3 signaling is not or only moderately activated. Our data suggest that inactive STAT3 acts either directly or indirectly as a negative regulator of COX-2, a feature that will be explored in future studies.”*

**Page 15, line 342:**

*“This deregulated arachidonic metabolism results in enhanced PGD_2_ production in cardiomyocytes which could also be confirmed in vivo as the treatment of CKO male mice with the COX-inhibitor Ibuprofen lowered the PGD_2_ secretion in CKO-CM. Thus, we provide evidence that enhanced COX-2 and sex-specific reduced HPGD contributes to elevated PGD_2_ production in male failure prone and failing hearts with low STAT3 expression (Fig 7)”*

**Page 15, line 359:**

*“The white adipocyte formation by PGD_2_ is mediated by the DP2-receptor since it could be inhibited by the DP2 receptor antagonist BAY-u 3405 but not by the DP1 receptor antagonist BWA868C. Moreover, since the treatment of CKO mice with the DP2 receptor antagonist BAY-u 3405 reduced the enhanced adipocyte formation of CKO-CPC, the role of PGD_2_ via its DP2 receptor for the formation of WAT could also be confirmed in vivo.”*

**Page 18, line 430:**

*“Finally, the present data suggest that the COX inhibitor Ibuprofen or the DP2 receptor antagonist BAY-u 3405 may attenuate these remodeling process and with this reduce age-related heart failure specifically in males with impaired AR signaling.*

*Limitation to this study: Although, we could show that COX inhibition and more specifically inhibition of the PGD_2_ receptor DP2 prevents the enhanced white adipocyte formation of CPC in failure prone CKO male hearts, we have not performed chronic treatment with COX inhibitors or BAY-u 3405 to test whether they would impact on age-related heart failure of the DCM phenotype.”*

**Method section, page 19, line 455:**

*“Ibuprofen and BAY-u 3405 treatment of CKO mice*

*Male CKO mice in the age of 9-10 weeks were treated with Ibuprofen (10 mg/kg bodyweight, Ibuprofen sodium salt, Sigma-Aldrich, dissolved in drinking water) or BAY-u 3405 (10 mg/kg bodyweight, Ramatroban, medchemexpress, dissolved in drinking water) for two weeks. CKO control animals received drinking water.”*
